# Supplementary material for: Causal Relationship between Aging and Anorexia Nervosa: A White-Matter-Microstructure-Mediated Mendelian Randomization Analysis
Source: Biomedicines. 2024 Aug 16;12(8):1874. doi: 10.3390/biomedicines12081874 (PMC11351342; doi:10.3390/biomedicines12081874)
Supplement: Supplementary file 1 [file biomedicines-12-01874-s001.zip › Supplementary Figure/Figure S8/Leaveoneout plot of MR analysis for TL to ubm-b-1462.pdf]

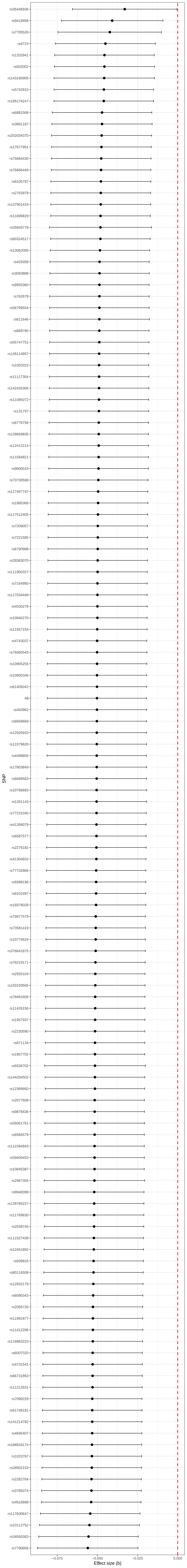

rs35446936
rs9419958
rs7705526
rs4724
rs1332941
rs932002
rs143190905
rs5742915
rs185174247
rs6881568
rs3891167
rs202034370
rs17677991
rs75664430
rs76666449
rs8105767
rs2763979
rs137901416
rs11699829
rs35640778
rs80324517
rs13062095
rs429358
rs3093888
rs9955360
rs762679
rs56799554
rs611646
rs869785
rs55747751
rs145114957
rs1003322
rs11117354
rs142426306
rs11085072
rs131797
rs6776756
rs139669835
rs12412214
rs11584821
rs9600019
rs73730598
rs117407747
rs1985369
rs117512405
rs7209057
rs7221585
rs6790988
rs28363070
rs111950327
rs7164950
rs117034449
rs4530278
rs10840270
rs11557154
rs4743037
rs76065543
rs10905255
rs10805346
rs61405042
All
rs450962
rs6659669
rs12925933
rs11579626
rs4498805
rs17803849
rs6669563
rs10768683
rs1291143
rs77231040
rs41269079
rs6587577
rs2276182
rs41304832
rs77732866
rs9398196
rs8102497
rs16978028
rs79977579
rs73581419
rs10774624
rs376641875
rs76219171
rs2555104
rs150150565
rs78491606
rs11426156
rs1957937
rs2230590
rs871134
rs1907702
rs6536702
rs144204502
rs12369950
rs2977608
rs9878436
rs56061761
rs6584579
rs112394943
rs59409453
rs10845387
rs2967355
rs9940099
rs139795227
rs11769630
rs2538745
rs111527438
rs12451892
rs939916
rs80116508
rs12932179
rs6590343
rs2056726
rs11991877
rs11412296
rs116863223
rs6007020
rs4731541
rs66731853
rs11212631
rs7099229
rs61748181
rs141214782
rs4695407
rs188918174
rs1023767
rs28502153
rs2282764
rs3785074
rs4616688
rs117630647
rs10112752
rs34550383
rs7790856
SNP
−0.075
−0.050
−0.025
0.000
Effect size (b)
